# Myogenin Regulates Exercise Capacity but Is Dispensable for Skeletal Muscle Regeneration in Adult *mdx* Mice

**DOI:** 10.1371/journal.pone.0016184

**Published:** 2011-01-14

**Authors:** Eric Meadows, Jesse M. Flynn, William H. Klein

**Affiliations:** 1 Department of Biochemistry and Molecular Biology, The University of Texas MD Anderson Cancer Center, Houston, Texas, United States of America; 2 Graduate Training Program in Genes and Development, The University of Texas School of Biomedical Sciences at Houston, Houston, Texas, United States of America; University of Dayton, United States of America

## Abstract

Duchenne muscular dystrophy (DMD) is the most prevalent inherited childhood muscle disorder in humans. *mdx* mice exhibit a similar pathophysiology to the human disorder allowing for an in-depth investigation of DMD. Myogenin, a myogenic regulatory factor, is best known for its role in embryonic myogenesis, but its role in adult muscle maintenance and regeneration is still poorly understood. Here, we generated an *mdx:Myog* ^flox/flox^ mouse harboring a tamoxifen-inducible Cre recombinase transgene, which was used to conditionally delete *Myog* during adult life. After tamoxifen treatment, three groups of mice were created to study the effects of *Myog* deletion: *mdx:Myog* ^flox/flox^ mice (*mdx*), *Myog* ^flox/flox^ mice (wild-type), and *mdx:Myog* ^floxΔ/floxΔ^:Cre-ER mice (*mdx:Myog*-deleted). *mdx:Myog*-deleted mice exhibited no adverse phenotype and behaved normally. When run to exhaustion, *mdx:Myog*-deleted mice demonstrated an enhanced capacity for exercise compared to *mdx* mice, running nearly as far as wild-type mice. Moreover, these mice showed the same signature characteristics of muscle regeneration as *mdx* mice. Unexpectedly, we found that myogenin was dispensable for muscle regeneration. Factors associated with muscle fatigue, metabolism, and proteolysis were significantly altered in *mdx:Myog*-deleted mice, and this might contribute to their increased exercise capacity. Our results reveal novel functions for myogenin in adult muscle and suggest that reducing *Myog* expression in other muscle disease models may partially restore muscle function.

## Introduction

Duchenne muscular dystrophy (DMD) is the most prevalent childhood muscle disorder in humans, affecting 1 in 3,500 newborn males [1]. DMD is a chronic muscle-wasting disease that leads to progressive muscle weakness and atrophy. It is caused by mutations within the dystrophin gene, a critical structural component of the muscle cell membrane [2]. The physiologic symptoms of DMD become apparent within the first few years of life, and muscle strength quickly deteriorates by puberty usually resulting in early death. Common diagnoses and signs of DMD include increased blood creatine kinase (CK) concentrations, increased rates of myofiber regeneration, centrally located myofiber nuclei, mitochondrial swelling, and ultimately myofiber necrosis [3]. Animal models for DMD include the *mdx* mouse, which has been studied extensively to better understand the molecular basis of the disease and to develop therapeutic strategies that might be applicable to humans [4], [5], [6], [7], [8], [9], [10].

As mentioned above, DMD is associated with muscle metabolism dysfunction and muscle weakness. Neuronal nitric oxide synthase (nNOS) is a dystrophin-associated protein found near the muscle sarcolemma, and its expression is greatly reduced in *mdx* mice [11]. Recently, investigators have shown that the loss of interaction between nNOS and phosphofructokinase (PFK) contributes to glycolytic dysfunction and increased fatigability in muscular dystrophy [12]. The forced expression of nNOS in *mdx* mice enhances muscle endurance and glycogen metabolism [12]. These experiments provide important mechanistic insights into why *mdx* mice, as well as DMD patients, experience progressive muscle weakness associated with muscle atrophy.

Nonhuman mammalian muscle fibers are broadly divided into subtypes based on their expression of different myosin isoforms: type-I, type-IIa, IIx/d and type-IIb fibers [13]. Type-I and type-IIa fibers are primarily oxidative and confer an increased exercise endurance with a high resistance to muscle fatigue during aerobic exercise. Type-IIb/type-IIx fibers are glycolytic and allow for anaerobic sprinting or short bursts of strength but fatigue more quickly. Type I fibers are commonly referred to as “slow twitch,” whereas type II fibers are known as “fast twitch.” Glucose is a major energy source for all muscle metabolisms, but glycolytic fibers are particularly dependent on glucose for energy production [13], [14]. Glucose stored as glycogen within skeletal muscle fibers is utilized to quickly meet the demands of high-intensity exercise. Fatty acids are the major source of energy utilized during low-intensity exercise. Lactate is a by-product of anaerobic metabolism and its blood concentration rises as exercise intensity increases [15].

The maintenance and regeneration of skeletal muscle are orchestrated by the 4 basic helix-loop-helix myogenic regulatory factors: MyoD, Myf5, MRF4, and myogenin. Within the last 20 years, *Myog* has been the subject of many studies that have demonstrated its diverse and relevant functions from embryonic development through adult life. Myogenin is required for embryonic skeletal muscle development and for achieving normal body size after birth [16], [17], [18]. Myogenin is primarily expressed in slow-twitch/oxidative myofibers, and when overexpressed, it has been shown to induce a shift from glycolytic to oxidative metabolism [19], [20]. Recently, myogenin was shown to transcriptionally activate the E3 ubiquitin ligases Fbxo32 and Trim63 following muscle denervation, and the loss of myogenin during adult life confers a resistance to denervation-induced muscle atrophy [21]. Moreover, recent results from our laboratory indicate that myogenin plays a role in regulating skeletal muscle metabolism and exercise capacity during adult life [22].

In this study, we sought to determine whether the deletion of myogenin in adult *mdx* mice would alter *mdx* muscle regeneration and exercise endurance capacity. Because myogenin is involved in muscle growth and regeneration in adult life, we hypothesized that deleting *Myog* in *mdx* mice would greatly exacerbate their muscle fatigue and wasting condition. Surprisingly, the absence of myogenin appeared to have no effect on muscle regeneration. Moreover, the exercise endurance of *mdx:Myog*-deleted adult mice was substantially improved compared to that of *mdx* control mice and was nearly equal to that of wild-type mice. This favorable improvement in exercise capacity was associated with alterations in nNOS and Fbxo32 expression, suggesting a molecular pathway responsible for improving muscle fatigue resistance.

Current therapies and treatments for DMD, such as corticosteriods and orthotics, are limited and only serve to delay the onset of this progressive muscle disease [23]. New therapeutic strategies aim to treat DMD at the molecular level, such as gene replacement therapy and exon skipping [24]. Our results reveal novel functions for myogenin in adult muscle and suggest that reducing *Myog* expression in other muscle disease models may partially restore muscle function.

## Results

### *Myog* is not required for the survival of *mdx* mice

**Figure 1:**
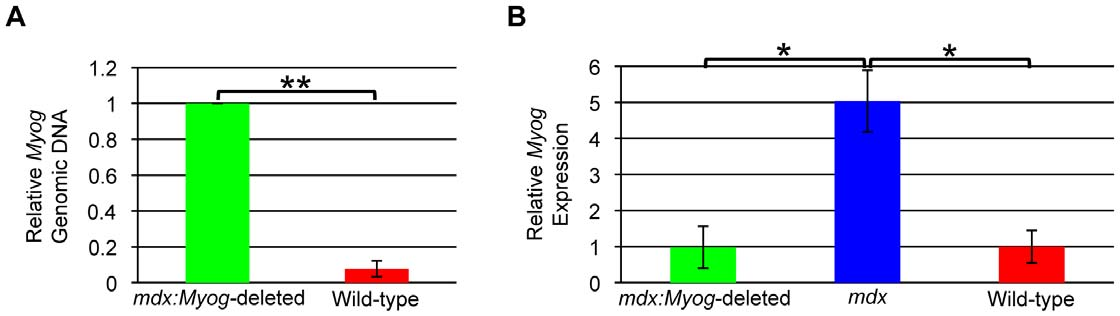
Reduced *Myog* genomic DNA and transcripts in *mdx:Myog*-deleted mice. (A) PCR genotyping reveals an efficient deletion (92%) of Myog genomic DNA in adult *mdx:Myog*-deleted mice (**p<0.001). (B) Reduced abundance of *Myog* transcripts in adult *mdx:Myog*-deleted mice. *Myog* transcript levels in *mdx* mice were increased by 5-fold over those in wild-type mice (*p<0.05). *Myog* transcript expression in *mdx:Myog*-deleted mice was equal to that of wild-type mice. (all groups, n = 3 ). Error bars represent 1 standard deviation.

Earlier, we hypothesized that deleting *Myog* in *mdx* mice would greatly exacerbate their muscle fatigue and wasting condition. To better understand the role of myogenin in adult skeletal muscle maintenance and regeneration and to test our hypothesis, we interbred mice carrying a floxed *Myog* allele and a tamoxifen-inducible Cre recombinase transgene (*Myog*-deleted mice), which were generated as previously described [17], with *mdx* mice (mice carrying the DMD*^mdx^* allele). Wild-type (*Myog^flox/flox^*) and *mdx* mice in the same mixed genetic background were also generated as controls. Cre-expressing adult mice (8–12 wk old) were rendered *Myog*-deficient with a single intraperitoneal (IP) injection of tamoxifen. The efficiency of *Myog* genomic deletion was approximately 92% (Figure 1A). Transcript expression analysis using reverse transcriptase-quantitative polymerase chain reaction (RT-qPCR) showed that induced expression levels of residual *Myog* in *mdx:Myog*-deleted mice were similar to basal expression levels of *Myog* in wild-type mice. Moreover, *Myog* transcript levels in *mdx* mice were 5 times higher than in wild-type mice (controls) (Figure 1B). This is expected given that *mdx* mice continuously regenerate their damaged muscle. *mdx*:*Myog*-deleted mice exhibited normal behavior, fed normally, and maintained normal body weight during a 1.5-year observation period (data not shown). These results indicate that *Myog* was not required for the survival of *mdx* mice.

### *mdx*:*Myog*-deleted mice exhibit an enhanced capacity for exercise compared to *mdx* mice

**Figure 2:**
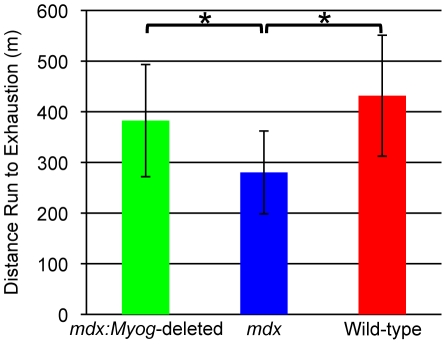
Enhanced exercise endurance capacity in *mdx:Myog*-deleted mice during the high-intensity exercise regimen. *mdx:Myog*-deleted mice (n = 14) over the course of 3 consecutive days of running ran 37% more than *mdx* mice (n = 14) and 11% less than wild-type mice (n = 14) under high-intensity exercise conditions. Error bars represent 1 standard deviation (*p<0.05).

Because the loss of myogenin in *mdx* mice did not result in any overt phenotypes and because our recent results showed that *Myog*-deleted mice have an enhanced capacity for exercise [22], we performed similar experiments with *mdx* and *mdx:Myog*-deleted mice. Both groups were run on a treadmill until they reached exhaustion using a high-intensity exercise regimen designed to test their exercise endurance capacity. Over the course of 3 consecutive days of running, *mdx:Myog*-deleted mice ran 37% more than *mdx* mice, but 11% less than wild-type mice before reaching exhaustion (Figure 2). These results demonstrate that the deletion of *Myog* increased the exercise endurance capacity of *mdx* mice.

### The absence of myogenin alters blood metabolite concentrations during exhaustive exercise

**Figure 3:**
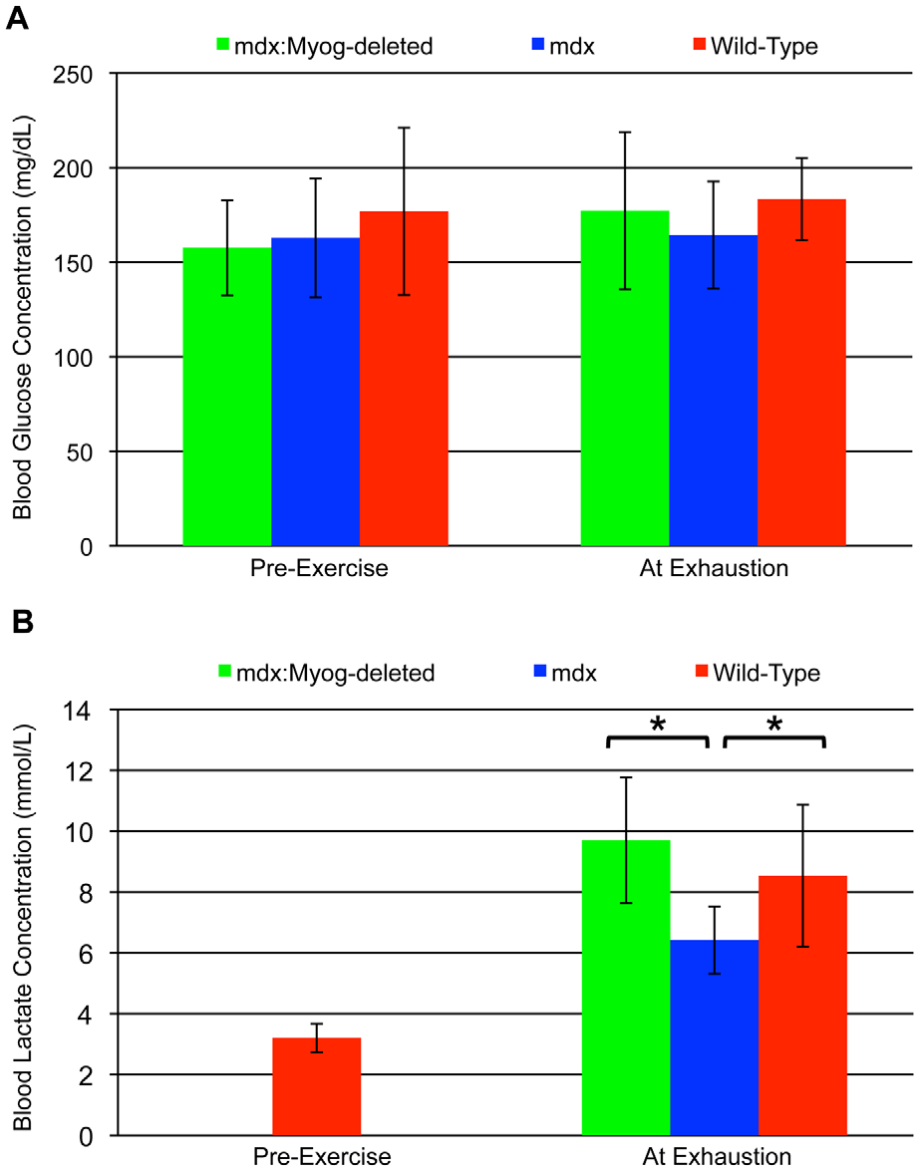
Normal blood glucose but increased blood lactate levels in *mdx:Myog*-deleted mice after high-intensity exercise. (A) Blood glucose levels in *mdx*, *mdx:Myog*-deleted, and wild-type mice (all groups, n = 14) after running to exhaustion under high-intensity exercise conditions. Baseline (pre-exercise) blood glucose levels were approximately the same for the 3 test groups, ranging from 158 to 177 mg/dL. At exhaustion, blood glucose levels remained virtually unchanged for all groups. (B) Blood lactate levels were significantly elevated for the 3 test groups at exhaustion compared to wild-type pre-exercise values. Blood lactate levels at exhaustion in wild-type (n = 10) and *mdx:Myog*-deleted (n = 12) mice were significantly higher than those of *mdx* mice (n = 11). Error bars represent 1 standard deviation (*p<0.05).

We previously found that deletion of *Myog* enhanced exercise endurance capacity and that this was associated with alterations in blood metabolite concentrations [22]. To investigate whether this phenomenon occurred in *mdx:Myog*-deleted mice, we determined their pre- and post-exercise metabolic profiles. Pre-exercise blood glucose concentrations were approximately equal between wild-type, *mdx*, and *mdx:Myog*-deleted mice. Moreover, in the 3 groups, these concentrations were virtually unchanged at exhaustion when compared to their pre-exercise levels (Figure 3A).

Lactate is a by-product of glycolysis, and increased blood lactate levels at exhaustion are a commonly used indicator of glycolytic metabolism, whereas basal blood lactate levels at exhaustion are indicative of oxidative metabolism. Blood lactate levels at exhaustion were increased in all 3 groups as compared to wild-type pre-exercise values. However, the blood lactate concentration level in *mdx:Myog*-deleted mice was 51% greater than *mdx* mice and 14% greater than that in wild-type mice (Figure 3B). These results show that *mdx:Myog*-deleted mice possessed a higher blood lactate concentration threshold than *mdx* and wild-type mice before reaching exhaustion.

### *Myog* is not required for muscle regeneration in adult *mdx* mice

**Figure 4:**
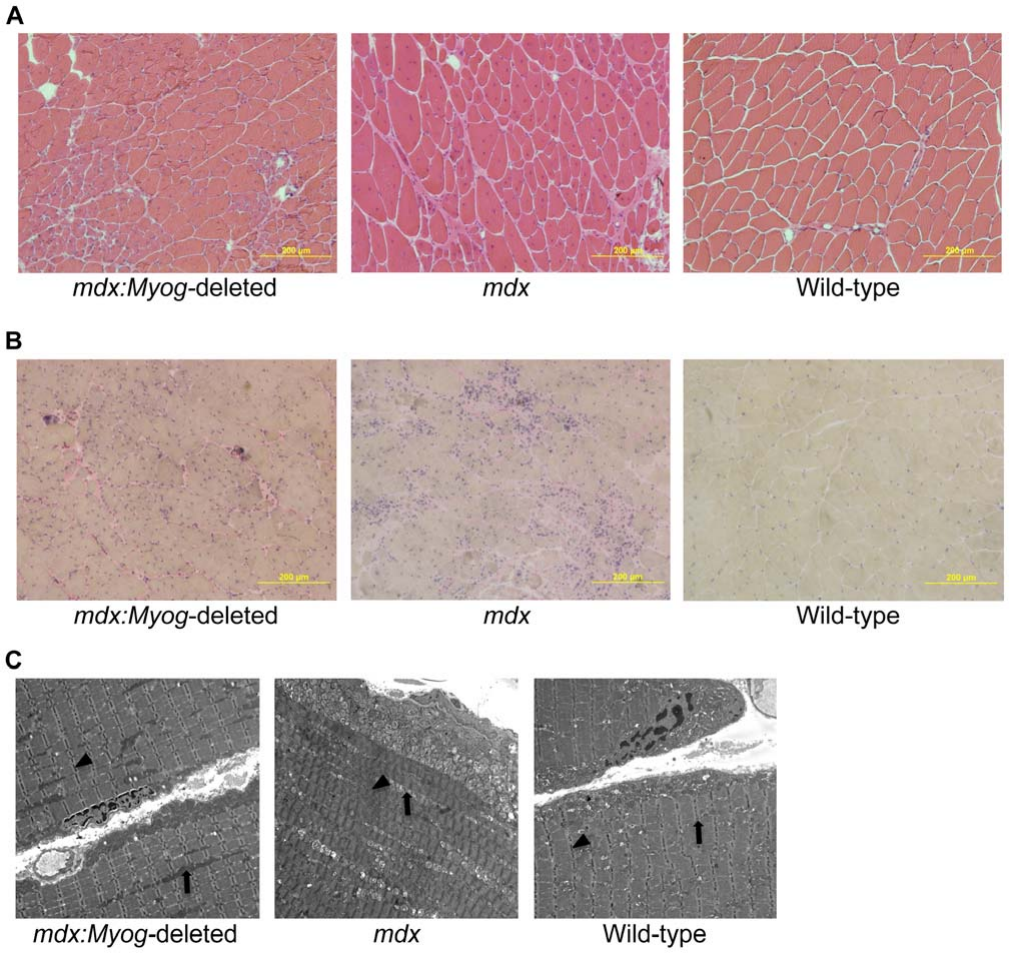
Histologic evaluation of *mdx*, *mdx:Myog*-deleted, and wild-type hindlimb muscle. (A) Hematoxylin and eosin-stained *mdx and mdx:Myog*-deleted muscle sections both displayed classic characteristics of regeneration, which include wide variations in myofiber diameter and centrally located nuclei, that were not observed in wild-type samples. (B) Van Gieson staining for collagen and elastin revealed similar levels of fibrosis in *mdx and mdx:Myog*-deleted muscle sections that were not observed in wild-type samples. (C) Ultrastructural analysis with TEM showed that *mdx:Myog*-deleted muscle sections have reduced mitochondrial swelling when compared to *mdx* samples. Arrows point out mitochondria. Arrow heads point out sarcomeric Z-lines; note the abnormal Z-lines in *mdx* muscle.

**Figure 5:**
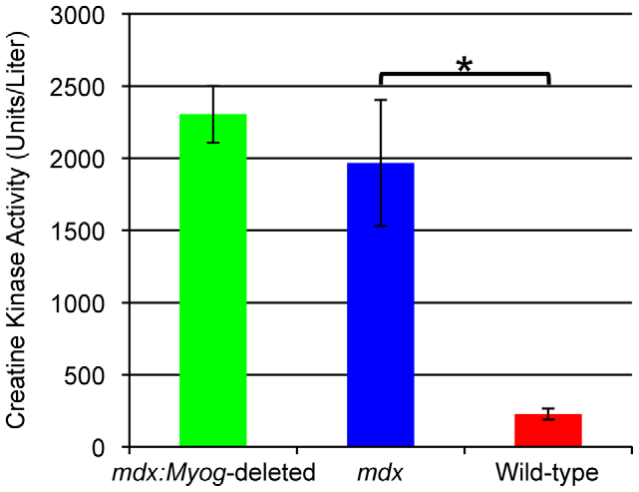
Elevated blood CK levels in *mdx* and *mdx:Myog*-deleted mice. A biochemical assay was used to determine the activities of CK in *mdx* (n = 10), *mdx:Myog*-deleted (n = 9), and wild-type (n = 6) mice. *mdx* mice exhibited an elevated level of CK prior to exercise compared to that of wild-type mice. Similarly, *mdx:Myog*-deleted mice showed elevated levels of CK prior to exercise. Error bars represent 1 standard deviation (*p<0.05).

Histology revealed that the muscle morphology of *mdx:Myog*-deleted mice was very similar to that of *mdx* mice. In both genotypes, we observed classic signs of muscle regeneration, such as myofibers with widely varying diameters containing centrally located nuclei (Figure 4A). Morphometric analysis revealed similar average cross-sectional areas for myofibers from both genotypes (data not shown). Van Gieson staining for collagen and elastin also revealed similar patterns of mild fibrosis in *mdx* and *mdx:Myog*-deleted mice (Figure 4B). To analyze the muscle ultrastructure, we used transmission electron microscopy (TEM). TEM analysis revealed signs of mitochondrial swelling in both *mdx* and *mdx*:*Myog*-deleted mice (Figure 4C); a visual inspection of the TEM sections showed the pathology to be somewhat worse in *mdx* mice than in *mdx*:*Myog*-deleted mice, as shown by abnormal, less well-defined sarcomeric Z-lines and swollen mitochondria (Figure 4C). To determine if the absence of *Myog* had an effect on the dystrophin-glycoprotein complex, we measured Creatine Kinase (CK) activity. Both *mdx* and *mdx*:*Myog*-deleted mice showed dramatically elevated CK levels compared to wild-type mice (Figure 5). The results demonstrate that deleting *Myog* in *mdx* mice did not reverse the pathologic effects of muscular dystrophy. Furthermore, we found that the deletion of *Myog* in *mdx* mice did not exacerbate their DMD, indicating that *Myog* is dispensable for muscle regeneration in adult *mdx* mice.

### Myofiber characteristics of *mdx:Myog*-deleted mice

**Figure 6:**
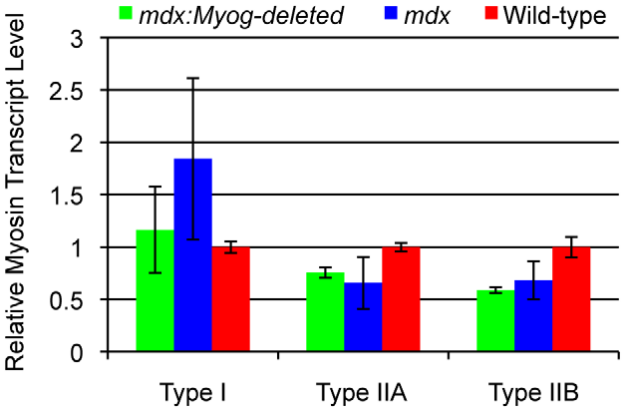
Myosin expression levels in *mdx*, *mdx:Myog*-deleted, and wild-type hindlimb muscle. No significant differences in transcript expression values, as assessed by RT-qPCR, for myosin isoforms type-I, type-IIa, and type-IIb were observed between *mdx* (n = 3) and *mdx:Myog*-deleted (n = 3) mice. Slight but significant alterations in myosins type-IIa and type-IIb were detected between wild-type (n = 3) and *mdx:Myog*-deleted mice. Error bars represent 1 standard deviation (*p<0.05).

**Figure 7:**
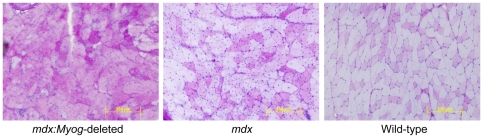
Similar muscle glycogen content in *mdx* and *mdx:Myog*-deleted mice. Glycogen staining showed similar levels of glycogen in *mdx* and wild-type gastrocnemius muscles, but suggested that *mdx:Myog*-deleted muscle had an increased trend towards glycogen content.

The deletion of *Myog* in *mdx* mice might be expected to cause alterations in muscle properties such as fiber type proportion and glycogen content. We performed RT-qPCR analysis to determine the transcript expression levels of myosin type-I, type-IIa, and type-IIb isoforms in medial and lateral gastrocnemius muscles taken from *mdx*, *mdx*:*Myog*-deleted, and wild-type mice. No differences were detected in the transcript expression levels of these myosin isoforms between *mdx* and *mdx*:*Myog*-deleted mice (Figure 6). We confirmed these RT-qPCR results by histological staining for myosin ATPase activity (data not shown). Glycogen histochemical staining of gastrocnemius muscle revealed a qualitative increase in glycogen content in *mdx*:*Myog*-deleted muscle compared to that of *mdx* and wild-type muscle (Figure 7A). We attempted to quantify these results by determining glycogen levels in whole muscle homogenates using a biochemical assay, but were unable to reliably detect any significant differences (data not shown). The results indicate that the absence of myogenin in *mdx* mice did not cause alterations in myosin isoform distribution or glycogen content. Therefore, the enhanced exercise endurance capacity that we observed in *mdx:Myog*-deleted mice compared to that in *mdx* mice was unlikely due to changes in their muscle properties.

### Expression levels of genes regulating muscle fatigue, metabolism, and proteolysis are altered in *mdx:Myog*-deleted mice

**Figure 8:**
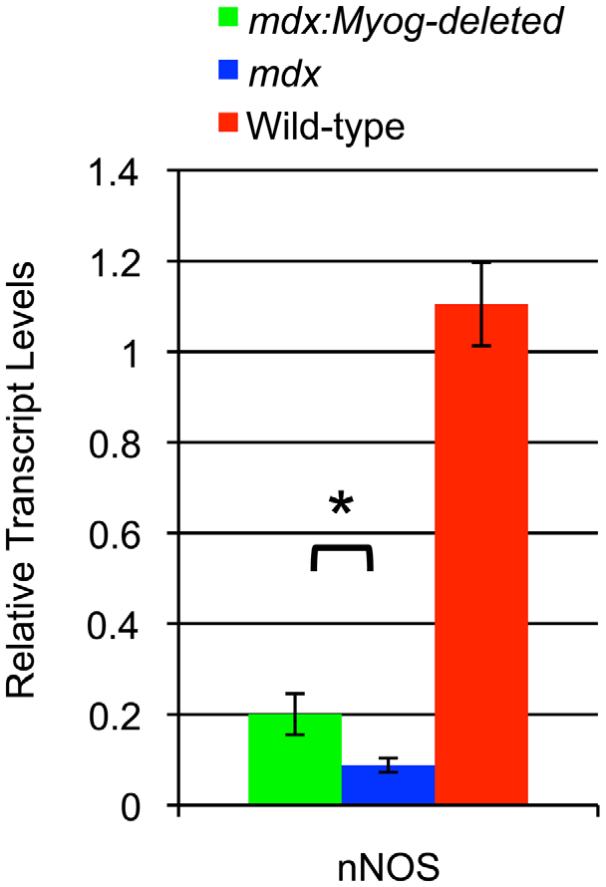
Altered expression of nNOS in *mdx*, *mdx:Myog*-deleted, and wild-type hindlimb muscle. RT-qPCR analysis demonstrated that transcript levels of nNOS were reduced in *mdx* mice (n = 3). Expression of nNOS was increased 2.2-fold in *mdx:Myog*-deleted mice (n = 3) compared to that in *mdx* mice. Error bars represent 1 standard deviation (*p<0.05).

To begin elucidating the mechanisms that are responsible for the enhanced exercise performance observed in *mdx:Myog*-deleted mice, we determined the transcript levels of genes whose expression might be expected to change in the absence of myogenin. Using RT-qPCR, we determined the transcript levels of genes that regulate muscle development, metabolic processes, and muscle proteolysis, namely, Myf5, MyoD, Mrf4, nNOS, PFK, Hdac4, glycogen synthetase, and muscle glycogen phosphorylase. In the light of recent findings showing that *Trim63* and *Fbxo32* are regulated by myogenin and promote muscle proteolysis and atrophy, we examined the expression of these genes [21]. Of these, the expression of Fbxo32 was significantly decreased 1.2 fold (data not shown) while the expression of nNOS increased 2.2 fold in gastrocnemius muscle of *mdx:Myog*-deleted mice compared to that of *mdx* mice (Figure 8). Changes in nNOS expression might have contributed to the enhanced exercise endurance capacity seen in *mdx:Myog*-deleted mice over their *mdx* counterparts. These alterations in gene expression suggest a mechanistic basis for this observed physiologic phenomenon.

## Discussion

Myogenin is thought to play a critical role in the maintenance and regeneration of adult skeletal muscle, but only a few studies supporting this view have been published. The discovery that *Myog* is required during embryonic mouse muscle development initially supported this view [16], and a number of subsequent studies have further suggested a role for *Myog* in adult muscle maintenance and regeneration [18], [19], [20], [25], [26], [27]. Recently, we developed an efficient method for conditionally deleting *Myog* during adult life [22]. Here, we applied this method to determine if the deletion of *Myog* had an effect on mice that were also deficient in dystrophin. Although the deletion of *Myog* before the onset of dystrophy may be of interest, we chose to delete *Myog* from dystrophic adult mice to avoid the growth defects observed in Myog-deleted mice [17]. The deletion of *Myog* in adult *mdx* mice also allowed us to clearly define the function of myogenin in adult *mdx* muscle. While the prevailing view of *Myog* would suggest an essential role for *Myog* in the maintenance and regeneration of *mdx* muscle, our results demonstrated that the absence of *Myog* in *md*x mice did not affect their behavior or mobility for up to 1.5 years following deletion of *Myog*. These results are consistent with our previous results showing that *Myog* is dispensable for skeletal muscle maintenance and regeneration during adult life [17], [18] [22].

Because *Myog* expression is activated upon muscle injury to help facilitate muscle regeneration and repair [28], and *mdx* mice exist in a constant state of muscle regeneration, we anticipated that *Myog* transcripts would be upregulated in muscle from *mdx* mice to help facilitate muscle regeneration. As expected, *Myog* transcript expression levels were dramatically increased in *mdx* mice but not in *mdx:Myog*-deleted mice. As skeletal muscle stem cells are activated to repair and fuse with existing muscle fibers, *Myog* expression increases in the latter stages of differentiation. These compensatory events result in muscle hypertrophy, which is characterized by increases in muscle mass, fiber count, centrally located nuclei, and distribution of the fiber calibers in *mdx* mice [10], [29], [30]. Our results also confirm previously published results demonstrating that *Myog* expression is elevated in *mdx* mice [31]. The efficient deletion of *Myog* and its subsequent downregulation in *mdx* mice did not produce any noticeable adverse effects, indicating that *Myog* is not required for survival or progression of muscular dystrophy. Our results demonstrate that when subjected to vigorous exercise, *mdx* mice exhibit a substantial reduction in exercise endurance capacity compared to wild-type mice. However, when *mdx:Myog*-deleted mice were subjected to vigorous exercise, they ran much farther than the *mdx* mice and approached distances ran by wild-type mice. These results are consistent with our previous findings that *Myog*-deleted mice have an enhanced capacity for exercise [22] and indicate that *mdx* mice, like wild-type mice, actually benefited from myogenin's absence.

In our previous study, we found that the deletion of *Myog* in adult mice enhanced their exercise endurance by altering their skeletal muscle metabolism as indicated by increased oxygen consumption and alterations in blood metabolite concentrations during exercise [22]. In our current study, we found that, at exhaustion, *mdx:Myog*-deleted mice had blood glucose levels similar to those of wild-type and *mdx* mice. This was expected because the high-intensity exercise regimen was designed to employ glycolytic metabolism, which primarily utilizes liver and muscle glycogen as a source for glucose. Mice subjected to high-intensity exercise often reach exhaustion before metabolizing substantial amounts of liver glycogen, thus leaving blood glucose concentrations at normal levels. The lack of observable blood glucose depletion in *mdx:Myog*-deleted mice suggests that the inherent muscle weakness in *mdx* mice limits their exercise endurance capacity, thus allowing their blood glucose concentration at exhaustion to remain at normal, pre-exercise levels.

Blood lactate concentrations were elevated in *mdx:Myog*-deleted mice compared to the *mdx* mice following high-intensity exercise. These results suggest that the loss of myogenin in *mdx* mice conferred an increased lactate threshold before reaching exhaustion. Recent studies have shown that, contrary to prevailing views, lactate production may actually retard muscle acidosis [32], [33], [34]. The conversion of pyruvate to lactate by lactate dehydrogenase also produces NAD+. This maintains the NAD+/NADH cytosolic redox potential, which promotes a substrate flux through glycolysis and ultimately allows for the continued production of ATP from glycolysis [35]. Thus, increased blood lactate levels may contribute to the observed increase in exercise endurance capacity in *mdx:Myog*-deleted mice.

*mdx* mice undergo muscle degeneration due to the absence of dystrophin at the sarcolemma [8], [9], [10], [11]. These muscle regeneration defects are readily apparent after bouts of intense exercise and generally result in chronic muscle weakness. Given that *mdx:Myog*-deleted mice exhibited an enhanced exercise endurance capacity, we investigated whether their muscles displayed signs of improved muscle regeneration. We found no remarkable changes in the muscle histopathology of *mdx:Myog*-deleted mice compared to that of *mdx* mice. Characteristic signs of muscle regeneration, such as myofibers containing centrally located nuclei and wide variations in fiber diameter, were observed in both *mdx:Myog*-deleted and *mdx* mice. *mdx* muscles are also particularly prone to fibrosis due to the increased production of connective tissue that is coincident with defective muscle regeneration and muscle atrophy [3]. We did not detect any differences in the abundance of fibrotic tissue from the muscles of *mdx:Myog*-deleted mice compared to those of *mdx* mice. However, on visual inspection, the muscles from *mdx:Myog*-deleted mice showed signs of reduced mitochondrial swelling and necrosis. Elevated CK levels due to the leakage of CK through the damaged sarcolemma are also characteristic of dystrophic muscle [36]. We observed dramatically elevated blood CK levels in both *mdx:Myog*-deleted and *mdx* mice compared to those in wild-type mice. Given that CK levels in *mdx:Myog*-deleted mice remained elevated after the deletion of *Myog*, one could conclude that the sarcolemmal defects normally associated with dystrophin deficiency were likely not repaired in these mice.

Although *mdx:Myog*-deleted mice displayed enhanced exercise endurance capacity than *mdx* mice, their histopathology was similar to that of *mdx* mice. Unexpectedly, *mdx* mice did not display an increased penetrance of muscular dystrophy following the deletion of *Myog*. *mdx:MyoD*-null mice exhibit a severe dorsal-ventral curvature of the spine, become progressively less active, and incur premature death [37]. Our results show that the loss of myogenin had no effect on muscle regeneration, which suggests that myogenin is dispensable for this process in adult *mdx* mice.

In mammalian muscle, enhanced exercise endurance is typically associated with increases in type-I and type-IIa muscle fibers as well as increases in muscle glycogen content [38]. Although we found a relative shift from type-II to type-I myofibers in *mdx* mice, the deletion of *Myog* in *mdx* mice did not alter these proportions. Glycogen content levels showed an increasing trend in *mdx:Myog*-deleted muscle in our PAS staining. Because the qualitative analysis of glycogen content could not be confirmed with a more quantitative biochemical assay, we conclude that glycogen content was unchanged in whole-muscle homogenates. Nevertheless, this did not appear to influence the increase in exercise endurance of *mdx:Myog*-deleted mice or the possibility that muscle glycogen content increases locally. Based upon our recent findings that mice lacking myogenin exhibit increased glycogen utilization and oxygen consumption during exercise [22], *mdx:Myog*-deleted mice are also likely to have similar physiological changes that contribute to their enhanced exercise endurance. We did not detect any changes in the expression of muscle glycogen synthetase or glycogen phosphorylase between *mdx:Myog*-deleted and *mdx* mice. Thus, *mdx:Myog*-deleted mice exhibit a higher exercise endurance capacity than *mdx* mice, yet they display myofiber characteristics similar to those of *mdx* mice.

Dystrophin deficiency confers a complex pathophysiology in *mdx* mice due to the secondary loss of other factors associated with the dystrophin-glycoprotein complex [12]. In fact, 80–90% of all dystrophin-glycoprotein–associated factors are absent in *mdx* mice [11]. These factors are synthesized normally, but in the absence of dystrophin, they are unable to assemble or integrate into the sarcolemma correctly and are thus targeted for degradation [39]. One of these factors is nNOS. nNOS-deficient mice have been shown to be unable to oppose vasoconstriction, resulting in ischemia and increased fatigability [40], [41]. Furthermore, nitric oxide (NO) has been shown to regulate neuromuscular transmission [42] as well as to promote mitochondrial biogenesis [43] and glucose transporter type 4 expression and transport [44], suggesting that reduced NO production may contribute to the early onset of fatigue [45].

Recently, Wehling-Henricks et al. showed that a loss of interaction between nNOS and PFK contributes to glycolysis defects and increased fatigability in *mdx* mice [12]. Given these results, we sought to determine whether nNOS and PFK expression would be affected by the deletion of *Myog* in *mdx* mice. Although the upregulation of nNOS expression in *mdx:Myog*-deleted muscle was modest compared to that in wild-type muscle, it was considerably higher than that in *mdx* muscle. The increase in nNOS expression in *mdx:Myog*-deleted mice might therefore contribute to the reduced fatigability observed in these mice and could partially explain their enhanced exercise endurance capacity compared to that of *mdx* mice.

With the current study, we extended the relevance of the *mdx* mouse model for investigating human DMD by demonstrating that the exercise endurance capacity of *mdx* mice is considerably lower than that of wild-type mice. The decrease in exercise endurance capacity that we observed in *mdx* mice is similar to the muscle weakness typically observed in DMD patients and is likely caused by similar mechanisms. In *mdx:Myog*-deleted mice, the enhanced exercise endurance was associated with increased expression of nNOS, a known regulator of muscle fatigue and atrophy. Because we did not find any histopathologic differences between *mdx* and *mdx:Myog*-deleted mice, we speculate that the absence of myogenin may rescue *mdx:Myog*-deleted mice from their inherent glycolytic dysfunction and muscle weakness, allowing for increased muscle endurance. Our study's findings suggest that reducing the expression of *Myog* in *mdx* mice by means other than gene knockout could provide new directions for achieving partial restoration of muscle function in other disease models.

## Materials and Methods

### Ethics Statement

All experimental procedures described in this study followed the U.S. Public Health Service Policy of Humane Care and Use of Laboratory Animals and were approved by the Institutional Animal Care and Use Committee of The University of Texas MD Anderson Cancer Center (ACUF ID#: 01-92-00237).

### Generation of *mdx:Myog*-deleted mice

For this study, we interbred mice that were homozygous for the *Myog^flox^* allele and hemizygous for the CAGGCre-ER transgene [17], [46] with C57BL/10ScSn-*Dmd^mdx^*/J mice (Jackson Labs) [4], [47]. Thus, *mdx:Myog* ^flox/flox^ mice were from a mixed background of B6, B10, and 129 strains. A control strain in the same background but wild-type for *DMD* was also generated. Sequencing analysis was performed to differentiate between mice carrying the *Dmd^mdx^* allele(s) and wild-type mice (mdx genotyping protocol details may be obtained by contacting the corresponding author). After tamoxifen treatment, three groups of mice were created to study the effects of Myog deletion: *mdx:Myog* ^flox/flox^ mice (*mdx*), *Myog* ^flox/flox^ mice (wild-type), and *mdx:Myog* ^floxΔ/floxΔ^:Cre-ER mice (*mdx:Myog*-deleted). Efficient deletion of *Myog* genomic DNA in 8 to 12 week old mice was achieved by a single injection of tamoxifen dissolved in corn oil at a concentration of 10mg/40g body weight. Genomic DNA deletion is determined 2 weeks following the deletion of *Myog* via qPCR. Deletion is initially tested in tail clippings and later confirmed in muscle tissue. To measure deletion efficiency, we used ABI Power SYBR and the following primers: Myog-LoxP-Fwd (5-CCG GGT AGG AGT AAT TGA AAG GA-3) and Myog-LoxP-Rev (5- GCC GTC GGC TGT AAT TTG AT -3). An ABI 7500 Fast Real Time PCR System was used to perform qPCR analysis using default conditions (95°C×10 min, 40 cycles of [95°C×15s, 60°C×60s]). Unless otherwise stated, all analyses were performed on mice at 3 to 4 months of age.

### Maximal Exercise Endurance Capacity

Treadmill exercise regimes we derived from previously published protocols [48]. Adult wild-type and Myog-deleted mice were run on a rodent treadmill (Columbus Instruments Animal Treadmill: Exer 3/6). This treadmill is equipped with a rear electrical stimulus grid set to deliver 0.2 mA, an uncomfortable but not physically harmful shock. Mice were determined to have reached the point of exhaustion when they made contact with the grid for a period of 10 seconds. The stimulus was then shut off in the lane of the exhausted mouse. The high-intensity exercise regime consisted of a warmup period of 10 minutes at 10m/min on a ten degree incline. The speed was then increased by 2 m/min every 2 minutes while maintaining a 10° incline until exhaustion. Exercise duration, distance, and maximum speed were recorded at exhaustion.

### Determination of blood lactate and blood glucose concentrations

Blood lactate concentrations were measured using a Lactate Pro blood lactate test meter (Arkray USA, Edina, MN) in mice before exercise and within 30 s after reaching exhaustion. Blood glucose levels were determined using a Precision Xtra glucometer (Abbott Labs, Chicago, IL) in mice before exercise and within 30 s after reaching exhaustion. Tail-vein blood extraction was used to determine blood lactate and glucose levels immediately after exhaustion.

### Histology

Hind-limb muscles were dissected and flash frozen in LN_2_-cooled isopentane. Ten to 12 µm fresh-frozen sections were then cut on a Microm HM 560 Cryostat. H&E Staining was performed as described previously [17].

*Glycogen Stain*: The Periodic Acid-Schiff Staining System from Sigma-Aldrich was utilized for this assay. Sections were hydrated with deionized water, fixed with 10% neutral buffered formalin for 30 minutes, and then incubated in Periodic Acid Solution for 5 minutes at room temperature. Sections were then rinsed in distilled water and incubated in Schiff's Reagent for 15 minutes at room temperature. Slides were then washed in running tap water for 5 minutes and counterstained in Hematoxylin Solution for 90 seconds. Slides were again washed in running tap water, dehydrated in ascending alcohols, cleared with 2 exchanges of Xylene, and mounted.

*Fibrosis stain for Collagen and Elastin:* Muscle sections were hydrated with deionized water and then fixed for 30 minutes in neutral buffered formalin. Slides were then incubated in Working Elastic Stain Solution for 10 minutes (20 mL Hematoxylin Solution, 3 mL Ferric Cholride Solution, 8mL Weigert's Iodine Solution, 5 mL Deionized water). Slides were then washed in deionized water and incubated in Wokring Ferric Chloride Solution for 1 minute (3mL Ferric Chloride Solution, 37 mL deionized water). Sections were then washed in tap water followed by rinsing in 95% alcohol. Slides were then rinsed in deionized water, incubated in Van Gieson Solution for 5 minutes, rinsed in 95% alcohol, dehydrated in ascending alcohols, cleared with 2 exchanges of Xylene, and mounted.

### Transmission Electron Microscopy

Samples were fixed with a solution containing 3% glutaraldehyde plus 2% paraformaldehyde in 0.1 M cacodylate buffer, pH 7.3, for 1 hour. After fixation, the samples were washed and treated with 0.1% Millipore-filtered cacodylate buffered tannic acid, post-fixed with 1% buffered osmium tetroxide for 30 min, and stained en bloc with 1% Millipore-filtered uranyl acetate. The samples were dehydrated in increasing concentrations of ethanol, infiltrated, and embedded in LX-112 medium. The samples were polymerized in a 70 C oven for 2 days. Ultrathin sections were cut in a Leica Ultracut microtome (Leica, Deerfield, IL), stained with uranyl acetate and lead citrate in a Leica EM Stainer, and examined in a JEM 100 transmission electron microscope (JEOL, USA, Inc., Peabody, MA) at an accelerating voltage of 80 kV. Digital images were obtained using AMT Imaging System (Advanced Microscopy Techniques Corp, Danvers, MA).

### Biochemical determination of glycogen content

Soleus and extensor digitorum longus (EDL) muscles were harvested from wild-type, *mdx*, and *mdx:Myog*-deleted mice. An abbreviated protocol followed from MBL International Corporation (www.mblintl.com) includes the following steps: muscles were isolated and snap frozen in liquid nitrogen. Muscle was homogenized in dH_2_O, boiled for 5 minutes, and centrifuged at 13,000rpm for 5min. Glucoamylase hydrolysis enzyme mix was then added to the supernatants. A master mix of development buffer, development enzyme mix, and OxiRed probe were then added to the samples and left to incubate at room temperature protected from light for 30 minutes. Samples were then measured colorimetrically using a spectrophotometer at a wavelength of 570nm. Non-hydrolysis control readings were then subtracted from the experimental raw values, and glycogen concentration was determined by applying the final values to a standard curve.

### RNA isolation and reverse transcriptase-quantitative PCR

RNA was isolated from dounce-homogenized mouse skeletal muscle or liver using the Invitrogen Trizol kit. Reverse transcription reactions were performed used Applied Biosystems RNA to cDNA kits. Power SYBR green, TaqMan master mix, and Taqman gene expression assays were purchased from Applied Biosystems (Foster City, CA) and used to determine cDNA abundance for each gene analyzed. An ABI 7500 Fast Real Time PCR System was used to perform qPCR analysis using default conditions (95°C×10 min, 40 cycles of [95°C×15s, 60°C×60s]). Taqman hydrolysis primer and probe gene expression assays were ordered with the following assay IDs: Myog, Assay ID: Mm00446194_m1; Gapdh, Assay ID: Mm03302249_g; Myod1, Assay ID Mm00440387_m1; Myf5, Assay ID Mm00435125_m1; Mrf4, Assay ID Mm00435126_m1; myosin 2a, Assay ID: Mm00454982_m1; myosin, heavy polypeptide 7, cardiac muscle, beta, Assay ID: Mm00600555_m1; myosin, heavy polypeptide 4, skeletal muscle, Assay ID: Mm01332541_m1; muscle glycogen phosphorylase, Assay ID: Mm00478582_m1; glycogen synthase 1, muscle, Assay ID: Mm00472712_m1; trim63, Assay ID: Mm01185221_m1; Fbxo32, Assay ID: Mm00499523_m1; Hdac4, Assay ID: Mm01299557_m1; nNOS, Assay ID: Mm00435175_m1; Pfkfb3, Assay ID Mm00504650_m1.

### Determination of blood creatine kinase concentration

To determine blood creatine kinase concentration, the Creatine Kinase-SL Assay Kit was used (Cat. No. 326-10/30, Genzyme). Briefly, fresh, clear, unhemolysed blood was first collected via tail-vein extraction. The serum was next separated from the sample by centrifugation and cooled on ice. The serum was then mixed with buffer reagents from the kit (R1 and R2) and the change in absorbance was spectrophotometrically measured at 340nm until the change in absorbance was constant. The change in absorbance values were then converted to Creatine Kinase (U/L) using the formula supplied with the kit.

### Statistical analysis

Statistical analyses were performed using 2-tailed pooled Student's *t*-tests in Microsoft Excel 2008. *P*<0.05 was considered to be statistically significant.
